# Too young to die? How aging affects cellular innate immune responses to influenza virus and disease severity

**DOI:** 10.1080/21505594.2021.1939608

**Published:** 2021-06-21

**Authors:** Christopher M. Harpur, Mélanie A. Le Page, Michelle D. Tate

**Affiliations:** a Centre for Innate Immunity and Infectious Diseases, Hudson Institute of Medical Research, Clayton, Australia; b Department of Molecular and Translational Sciences, Monash University, Clayton, Australia

**Keywords:** influenza, aging, pathogenesis, host-pathogen interactions

## Abstract

Influenza is a respiratory viral infection that causes significant morbidity and mortality worldwide. The innate immune cell response elicited during influenza A virus (IAV) infection forms the critical first line of defense, which typically is impaired as we age. As such, elderly individuals more commonly succumb to influenza-associated complications, which is reflected in most aged animal models of IAV infection. Here, we review the important roles of several major innate immune cell populations in influenza pathogenesis, some of which being deleterious to the host, and the current knowledge of how age-associated numerical, phenotypic and functional cell changes impact disease development. Further investigation into age-related modulation of innate immune cell responses, using appropriate animal models, will help reveal how immunity to IAV may be compromised by aging and inform the development of novel therapies, tailored for use in this vulnerable group.

## Introduction

Influenza is a common, widespread infectious disease, estimated to be responsible for close to 400,000 annual deaths worldwide and substantial morbidity [1]. Influenza A virus (IAV) infections are often asymptomatic; however, common symptoms include fever, chills and headache. Viral pneumonia is a rare complication of IAV infection and occurs mainly in immunocompromised individuals with underlying medical conditions such as chronic heart or pulmonary diseases and comorbidities including those associated with obesity and smoking [2,3]. Severe IAV infections in humans are characterized by hyperinflammatory responses, as well as vascular leakage and pulmonary edema; hallmark pathological features of acute respiratory distress syndrome (ARDS) [4]. Additionally, IAV infection can commonly lead to subsequent secondary bacterial infection, which account for the majority of influenza-associated deaths in the elderly or during IAV pandemics [5,6]. Indeed, during the 2009 Swine H1N1 pandemic it was estimated that up to 55% of mortalities resulted from secondary bacterial pneumonia. With our increasing longevity, the human population is rapidly aging, with over 700 million people currently over the age of 65, a figure that is expected to double within 30 years [7]. This is particularly alarming as the elderly are routinely overrepresented in influenza-related fatalities and hospitalization and the risk of death increases with advancing age [1,8,9].

The innate immune system forms the first line of defense against IAV infection and is partially comprised of a heterogenous assortment of fast-acting immune cells, which collectively act to limit the dissemination of the virus and shape the character of the ensuing adaptive immune response. However, a dysregulated hyperinflammatory innate immune cell response can cause significant damage to the lung [10,11]. and IAV disease severity and successful recovery from infection is proposed to result from either efficient elimination of the virus or by tolerance of the infection and limiting the associated immunopathology [12]. Irregular immune cell function (immunosenescense) and the persistent low levels of systemic sterile inflammation (inflammaging) are consequences and common features of aging and positively correlate with increased susceptibility in older individuals, (>65 years old); however, how these age-associated changes affect innate immunity to IAV remains to be fully elucidated [13,14]. Due to limitations on studying IAV disease in humans, animal models of acute infection are heavily relied upon to examine important aspects of the disease and the immune response to it. Most of our knowledge stems from murine models of IAV infection owing to the relative ease that mice can be genetically manipulated, in addition to the prohibitive ethical and financial barriers associated with using higher organisms such as ferrets and non-human primates, to model this disease, particularly in the context of aging. Translation of findings from animal models to the clinic can be difficult, however, particularly from these heavily utilized mouse models of IAV infection. Notable issues include differences in IAV infection susceptibility between inbred laboratory mouse strains [15,16], which also possess a genetic-deficiency in a key antiviral factor normally present in mice and humans that shows reduced expression in monocytes from elderly humans [17,18]. Specifically, most laboratory mouse strains lack functional *Mx1* alleles the encode an interferon (IFN)-inducible influenza resistance factor (Mx1), which when restored can imbue mice with increased resistance to several respiratory viruses [18–20]. Nevertheless, across several different mouse strains and consistent with the poorer influenza disease outcomes in elderly humans, most reports indicate that aged mice (>16 months of age) exhibit increased lung tissue damage, increased inflammation, impaired viral clearance, increased weight loss and decreased resistance to IAV infection [21–30]. Similar observations have been made in IAV-infected non-human primates with increased levels of virus and proinflammatory interleukin (IL)-6 present in the lungs of older rhesus macaques compared to younger counterparts [31]. Other recent studies, however, have reported that aged C57BL/6 mice (16–30 months of age) are more resistant to mouse-adapted A/PR8/8/34 (PR8; H1N1) or A/California/04/09 (2009 pandemic H1N1) infection than young adult mice (6–9 weeks of age) [18,32]. These studies used a relatively low volume of IAV inoculum (20 μL), a seemingly important experimental distinction as in direct comparison, aged C57BL/6 inoculated with PR8 using this volume showed no age-associated changes in survival, while the same viral dose administered in 40 μL induced increased mortality only in the older mouse cohort [33]. Despite these inconsistencies, such models have provided valuable insight into the pathogenesis of influenza disease in aged organisms and as detailed below, how various facets of the innate immune system response to IAV infection are altered. Here, we review how aging affects the early stages of IAV infection as well as the innate cellular immune response it elicits and examine how these changes may contribute to the increased influenza-associated morbidity and mortality observed in the elderly human population.

### Influenza virus and early infection dynamics

Influenza viruses belong to the *Orthomyxoviridae* family, which is comprised of type A, B and C viruses. IAV cause most influenza epidemics in humans and have the potential to cause severe pandemics due to their ability to readily mutate and as such will be the focus of this review. While type B influenza viruses also circulate in the human population, they mutate at a significantly slower rate than IAV [34] and are generally associated with less severe epidemics. Type C influenza viruses induce mild disease in man and have not been associated with either epidemics or pandemics.

IAV is an enveloped virus consisting of a segmented negative-sense ribonucleic acid (RNA) genome surrounded by a lipid bilayer derived from the host cell plasma membrane. The single-stranded viral genome is organized into eight segments, each of which forms a distinct ribonucleoprotein (RNP) complex within the virion. The RNA segments encode for 11 viral proteins, 9 of which have been identified within the virion and 2 additional proteins, PB1-F2 and non-structural (NS1), that are expressed in infected cells [35]. The hemagglutinin (HA) and the neuraminidase (NA) glycoproteins are embedded in the viral envelope and form the characteristic surface spikes of the virus that are visible by electron microscopy [36]. IAVs are subtyped according to the serological reactivities of their HA and NA surface glycoproteins. To date, 18 avian and mammalian HA and 11 NA subtypes have been identified and while some avian subtypes (e.g. H5N1, H7N1, H7N3, H7N7 and H9N2) have sporadically infected humans, only 3 HA subtypes (H1, H2 and H3) and 2 NA subtypes (N1 and N2) are known to have circulated in the human population [37].

During the early stages of IAV infection, pathogen-associated molecular patterns are detected by pattern recognition receptors (PRR), including retinoic acid inducible gene-I (RIG-I)-like receptors and toll-like receptors (TLR), leading to the induction of proinflammatory signaling pathways. For example, TLR3 and TLR7 in the endosomal compartment and RIG-I in the cytosol recognize viral single-stranded RNA (ssRNA) or the ‘panhandle” structure of the (sub)genomic RNA/double-stranded RNA (dsRNA), respectively [10,38], leading to the activation of nuclear factor kappa-light-chain-enhancer of activated B cells (NF-κB), which mediates the production of proinflammatory cytokines and chemokines, such as interleukin (IL)-6, tumor necrosis factor (TNF)α, C-C motif chemokine ligand (CCL)2, pro-IL-18, and pro-IL-1β. Additionally, interferon regulatory factor (IRF)3 and IRF7 activation results in production of type I (IFNα and IFNβ) and III (IFNλ) IFNs, which play a major role by inducing the expression of hundreds of genes encoding proteins involved in limiting IAV infection and replication, as well as influencing cell migration, proliferation, differentiation and survival [39,40]. Activation of NOD-, LRR- and pyrin domain-containing protein 3 (NLRP3), a cytosolic PRR, during IAV infection facilitates inflammasome complex formation and enzymatic maturation of inactive precursors pro-IL-1β and pro-IL-18 into bioactive IL-1β and IL-18 [10,38]. Several changes to these signaling pathways have been associated with aging, which have recently been reviewed elsewhere [41] and therefore will not be the focus of this review. Rather, notable age-associated differences in the early cellular response to IAV infection and these signaling events, particularly changes in innate immune cell abundance, phenotype and function will be covered here.

Epithelial cells lining the respiratory tract and associated respiratory macrophages are the initial targets of IAV infection. Respiratory epithelial cells serve as the primary source of IAV replication, amplification and release of new virions [42–44], which largely results in cell death [10]. Infection is initiated via the IAV hemagglutinin (HA) protein binding to sialic acid on the cell surface of epithelial cells, which triggers endocytosis of the virion. Following fusion of viral and cellular membranes, viral RNP are released into the cytoplasm and enter the nucleus [45]. Once inside the nucleus, viral RNP undergo transcription and replication and progeny viral RNP are then exported to the cytoplasm. Viral proteins are translated, assembled and transported to the plasma membrane prior to the budding and the release of newly synthesized virions from the surface of infected cells. By contrast, entry into respiratory macrophages typically leads to destruction of the virus and apoptosis of the infected cell [42–44]. The early inflammatory response to IAV infection is largely determined by the spectrum of chemokines and cytokines released from respiratory epithelial cells and macrophages. IAV infection of primary respiratory epithelial cells and cell lines from various species results in the production of proinflammatory cytokines IL-6 and TNFα, type I IFNs and leukocyte-attracting chemokines CCL2, CCL5 and C-X-C chemokine ligand (CXCL)8 [46–53]. Macrophages/monocytes infected with IAV are also potent producers of proinflammatory cytokines IL-6 and TNFα, type I IFNs and chemokines CCL2, CXCL8 and CXCL10 [54–57]. The production of inflammatory cytokines following IAV infection is critical for the initial control of virus replication; however, aberrant cytokine responses can be detrimental to the host, with hyperinflammatory “cytokine storms” often associated with severe morbidity and mortality in infected humans [58–61]. The early cytokine and chemokine production elicited by IAV-infected respiratory epithelial cells and macrophages contributes to the activation, rapid recruitment and local expansion of several innate immune cells that have demonstrated roles in defense against, and recovery from, influenza disease. This includes the infiltration of neutrophils into the lung within 24 hours post-infection followed closely by the accumulation of natural killer (NK) cells, inflammatory macrophages and dendritic cells (DC) in the airways [62,63] and subsequently, the activation of local type 2 innate lymphoid (ILC2) cells, that can assist lung tissue repair [64–66].

**Figure 1.**
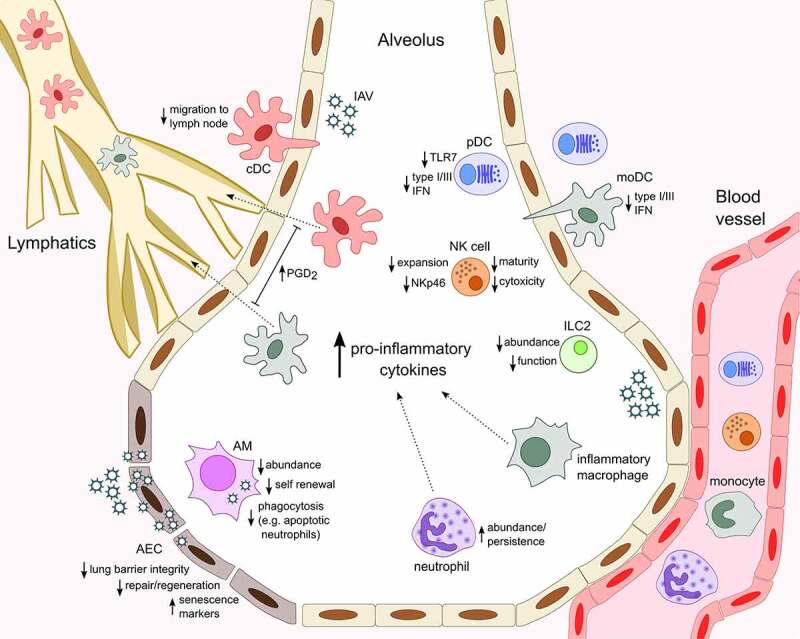
Age-associated changes in innate immune cell response to influenza infection

The production of high levels of cytokines during severe IAV infection is also thought to contribute to epithelial damage, leading to vascular leakage and pulmonary edema. This loss of epithelium integrity may increase the host’s susceptibility to secondary bacterial infection, which is a major complication associated with IAV infection and can be fatal, particularly in the elderly, whom may already have had reduced lung epithelial barrier function [5,6,67]. In the lung, Club (or Clara) cells are non-ciliated, non-mucous, secretory cells, which predominate in the bronchioles, whereas two forms of epithelial cells exist within the alveoli, namely alveolar epithelial cell (AEC) type I and type II. Club cells exhibit progenitor cell properties and are thought to mediate maintenance and repair of both the bronchiolar and alveolar epithelium following IAV infection [68,69]. Epithelial repair is a critical process for recovery from IAV infection and aging has been shown to be associated with a functional decline in stem and progenitor cells [70], potentially compromising tissue maintenance and repair. A study by Yin and colleagues suggested that there was no difference in the extent of damage and kinetics of regeneration by club cells between young (2–3 months) and aged (16–18 months) C57BL/6 mice following PR8 infection [69]. However, increased damage to type I and type II AEC and a delay in the regeneration of type II cells was found in aged mice. Additionally, AEC from 18–22-month-old C57BL/6 mice have been shown to produce higher levels of neutrophil-attracting chemokines CXCL1 and CXCL2 following PR8 infection, while no differences were noted in IL-1β and TNFα production (Figure 1) [30]. The authors proposed that this enhanced chemokine production was associated with an increased presence of senescent AEC in aged mice both before and after infection.

### Macrophages

Lung-resident interstitial macrophages (IM) and alveolar macrophages (AM) are phagocytic cells that reside in the airways and respond rapidly to invading microbes, including viruses. Located in the alveoli, AM have direct contact with our environment and area critical first line of defense against inhaled pathogens. Due to their location, AM can be collected relatively easily in bronchoalveolar lavage (BAL) fluid from humans and animals for analysis [42,43] and are thought to have a regulatory phenotype, working to limit excessive inflammation in the airways [71,72]. Both IM and AM express CD169 in humans and mice, are capable of local self-renewal from progenitor populations or after IAV infection, can be replenished from circulating monocytes [73–76], yet IM exist in the interstitium along with respiratory DC and are much less abundant than AM [77,78]. IM can be distinguished from AM by their lack of CD11c expression [79] and are generally less phagocytic but more effective at stimulating T cell proliferation [77,80].

As sentinels of the airways, AM are a primary target of influenza infection and are susceptible to infection by most IAV [81], with the highly virulent mouse-adapted PR8 strain being a notable exception [44,82,83]. Typically, infection of AM results in early virus-specific protein synthesis [84] but this is interrupted within a few hours and no infectious viral progeny are released prior to the infected AM undergoing apoptosis [42–44]. As such, IAV infection of mouse and human AM is generally considered to be abortive, although particular highly pathogenic H1N1 and H5N1 IAV can infect and replicate productively to some degree in murine and human macrophages [85]. As previously mentioned, human and murine macrophages respond to IAV infection via the production of an array of proinflammatory cytokines and chemokines, which act to limit virus spread and recruit inflammatory leukocytes to the lung. As IAV vary in their ability to infect AM [43,44], the cellular tropism of the virus could be a critical factor in determining the airway cytokine milieu during the early phase of infection, which in turn impacts the specific cell types recruited to the lung and the nature of the subsequent immune response. Additionally, the release of the damage-associated molecular pattern (DAMP) IL-33 by infected AM may be critically important for recovery from IAV infection [86,87]. The crucial role of respiratory macrophages *in vivo* following IAV infection has been illustrated in numerous depletion studies. In particular, treatment of mice with clodronate-loaded liposomes during infection with mild and virulent strains of IAV is associated with enhanced virus replication [44,88–92], increased inflammation, as well as alveolar leakage and pulmonary edema [44]. This method of depletion also eliminates other phagocytic cells in the lung, yet similar observations were made in IAV-infected mice after conditional ablation of CD169^+^ AM and IM [93].

IAV infection triggers the rapid recruitment of circulating leukocytes to the lung, dominated by neutrophils and monocytes, the latter of which being able to differentiate into macrophages and DC. Monocyte-derived inflammatory Ly6C^+^ macrophages infiltrate the lung in high numbers several days following IAV infection in response to CCL2 produced either by AEC or previously recruited inflammatory macrophages [47,63,94–96]. This latter source of CCL2, which sustains inflammatory macrophage recruitment to the lung requires intrinsic type I IFN signaling, which also appears to regulate infiltration of other leukocytes [94]. Indeed, mice lacking the type I IFN receptor (*Ifnar1^-/-^*), including strains with intact Mx1, more readily succumb to PR8 H1N1 and avian H7N7 IAV infection and possess reduced lung infiltrating inflammatory macrophages, increased numbers of neutrophils and elevated levels of the neutrophil-attracting chemokine CXCL1 in the lung [94,97]. Contradicting observations have been made in IAV-infected mice lacking key mediators of monocyte chemotaxis. In support of Ly6C^+^ inflammatory macrophages playing a protective role during severe IAV infection, another study demonstrated that PR8-infected mice deficient in CCL2 were found to be more susceptible to influenza disease [98]. In contrast, recruited Ly6C^+^ inflammatory macrophages have also been shown to contribute to lung damage and mortality during severe IAV infection as demonstrated by several studies using CCL2- or CCR2-deficient mice, which display improved survival rates following infection with PR8 H1N1 or avian H7N9 IAV [99–102].

Aging is generally accepted to result in increased basal levels of inflammatory mediators including cytokines and chemokines. IAV-infected aged mice have been shown to have elevated levels of TNFα, IL-1α, IL-6 and monocyte-attracting CCL2 while similarly, older rhesus macaques (20–24 years of age) infected with 2009 pandemic H1N1 IAV displayed significantly higher levels of IL-6 and CCL2 in their lungs compared with younger macaques (10–12 years of age) [22,26,31,103]. Interestingly, monocytes and macrophages from older individuals (65–89 years of age) produce similar amounts of IL-6 and IL-1β after *in vitro* infection with PR8 H1N1 compared to those from younger donors (20–30 years of age), yet type I IFN production, as well as the expression of IFN signaling molecules and IFN-stimulated genes were reduced [18]. In mouse studies, Wong and colleagues demonstrated that increased age is associated with reduced numbers of AM in the lungs of 22–24- month-old C57BL/6 and BALB/c mice both prior to and on day 6 following PR8 infection compared to younger (2–4 month-old) counterparts [25] (Figure 1). Macrophage deletion by clodronate-loaded liposome treatment was shown to decrease survival in both young and aged mice and retention of neutrophils in the lungs, suggesting macrophages play a critical role during IAV infection independent of age [25]. However, using adoptive transfer experiments and measuring lactate dehydrogenase release, the authors demonstrated that aging impairs the intrinsic ability of AM to limit lung damage during PR8 infection. Transcriptional analysis also revealed a downregulation of genes involved in cell cycle pathways such as metaphase checkpoint and chromosome separation, whereas CXCL8 and vascular endothelial growth factor signaling was enhanced with increasing age. Aging AM were also shown to exhibit a defect in phagocytosis of apoptotic neutrophils, as well as the expression of the phagocytic receptor CD204 [25]. Similar *in vitro* phagocytic defects were observed in AM from aged (26-monthold) C57BL/6 mice [104]. Significant IAV-induced depletion of AM has also been observed in young BALB/c mice after PR8 infection, yet Toapanta and colleagues reported a similar degree of macrophage accumulation in the lungs of 72–76-week-old and 12–16-week-old BALB/c mice until day 15 post-infection, at which point significantly higher numbers of lung macrophages persisted for at least a further 4 days in the older animals [22,105]. Upregulation of the activation marker CD40 on this unspecified lung macrophage population was delayed in these infected aged BALB/c mice, suggestive of an age-associated impact on their function [22]. A recent study found that aged AM from 18–20-month-old BALB/c mice exhibit decreased indoleamine-pyrrole 2,3-dioxygenase (IDO1)-dependent tryptophan metabolism following PR8 infection [26]. This deficiency correlated with increased lung viral load, tissue damage, proinflammatory IL-6 and IL-1β in aged mice, as well as reduced IL-10 and fewer regulatory T cells, that support recovery from IAV infection. Interestingly, *in vivo* treatment of IAV-infected young mice with an IDO1-inhibitor enhanced viral titers, inflammation and morbidity while treatment of aged mice with mitoquinol, a mitochondrial targeted antioxidant, restored tryptophan metabolism and reduced weight loss and levels of virus in the lung, demonstrating the importance of this pathway [26]. Of note, the fate of murine AM following IAV infection of mice appears to be more dependent on mouse strain rather than virus strain or inoculum dose. Specifically, Califano and colleagues recently reported that PR8 and 2009 pandemic H1N1 infection causes depletion of AM in BALB/c mice in an IFNγ-dependent manner, but only diminished AM phagocytic activity in C57BL/6 mice, a discrepancy that should be considered when examining AM in aged mice in future studies [106].

Finally, multiple studies have shown that AM from recently IAV-infected mice are either depleted, phenotypically altered and/or functionally paralyzed, resulting in reduced phagocytic capacity and increased susceptibility to secondary infection by opportunistic bacteria for weeks following the initial viral infection [6,105,107]. A similarly sustained phagocytic defect was observed in human monocytes from patients with trauma-induced inflammation and those recently cured of sepsis [107]. Therefore, the combination of this stunning effect of IAV infection on AM that may already be diminished in frequency and function together with the compromised lung epithelium associated with increased age, could help explain the increased susceptibility of elderly (>65 years old) IAV-infected individuals to secondary bacterial pneumonia [5,6,67].

### Dendritic cells

Dendritic cells (DCs) are a critical bridge between the innate and adaptive arms of the immune system. DC express an array of PRR such as TLR and C-type lectins, which allow detection of invading pathogens and changes in the local environment, and this information is conveyed to nearby lymphoid organs to prime CD4^+^ and CD8^+^ effector T cell responses [108]. Presentation of antigen to T cells is the primary role of respiratory CD11c^+^ conventional (cDC) that migrate to the lung-draining mediastinal lymph node, whereas the principal function of plasmacytoid DC (pDC) is the production of type I IFNs. The main cDC subsets in the uninfected lung are the CD103^+^ and CD11b^+^ cDC, sampling environmental antigens proximal to the respiratory epithelial cells [109]. pDC are a minority DC subset in the lung and are found in the large conducting airways and lung interstitium [110]. These cells express high levels of endosomal TLR7, which senses virals sRNA and upon TLR7 stimulation, pDC produce vast amounts of antiviral type I IFNs (IFNα and IFNβ), which are important for recruiting leukocytes from the blood and shaping the immune response [111,112]. Following PR8 infection of BALB/c mice, circulating monocytes enter the lung and can differentiate into monocyte-derived DC (moDC), which together with the lung-resident CD103^+^ DC and CD11b^+^ DC can then traffic IAV antigens to the draining lymph node where they present viral antigen to naïve CD4^+^ and CD8^+^ T lymphocytes to facilitate viral clearance [113]. *In vivo* co-culture experiments have demonstrated that CD4^+^ T cell expansion on IAV antigen can be driven by all three DC subsets; however, CD103^+^ DC are the most efficient stimulators of CD8^+^ T cell responses; a finding supported by other groups [113–116]. Additionally, GeurtsvanKessel and colleagues illustrated that depletion of langerin^+^ CD11b^−^ respiratory cDC (CD103^+^ DC) during HKx31 H3N2 IAV infection resulted in increased clinical disease severity and delayed viral clearance, which correlated with a sharp decrease in virus-specific CD8^+^ T lymphocytes [117]. Following *in vitro* infection with A/Japan/305/57, murine CD103^+^ DC readily produce IL-12 and CXCL1, whereas CD11b^+^ DC secreted CCL5 [118]. During PR8 infection, CD11b^+^ DC express less CCR7 and do not migrate as efficiently to lymph nodes; however, *ex vivo* re-stimulation of DC from infected lung with synthetic dsRNA polyinosinic-polycytidylic acid (poly I:C) shows CD11b^+^ DC to a be potent producers of proinflammatory TNFα [116]. Respiratory cDC are also pivotal producers of antiviral type III IFN (IFNλ), which act directly upon lung epithelial cells, as evidenced by targeted depletion of CD11c^high^ cells in mice administered poly I:C being sufficient to completely abolish the TLR3-mediated IFNλ response [119,120]. In C57BL/6 mice, type III IFN were previously shown to be beneficial for viral mucosal immunity; however, more recent studies have suggested they can contribute to damage and hinder epithelium repair, which can facilitate bacterial superinfections [120–124].

pDC-derived type I IFN can induce apoptosis of infected cells and activate NK cells and CD8^+^ T cells during IAV infection [125]. Using BALB/c mice infected with respiratory syncytial virus for comparison, Jewell and colleagues observed that antibody-mediated depletion of pDC only resulted in a significant loss of type I IFN in the lungs of A/WSN/33 H1N1 IAV-infected animals [48]. The authors concluded that during IAV infection, pDC and lung epithelial cells are the main sources of type I IFNs. Similarly, anti-120G8-mediated depletion of pDC in PR8-infected BALB/c mice also resulted in significantly less IFNα in the lung as well as reduced weight loss and lung viral titers [126]. Interestingly, in the absence of pDC, AM, monocyte-derived macrophages and CD11b^+^ DC expanded in the lung with a greater proportion of each of these populations producing proinflammatory mediators TNFα and IL-6 [126]. Despite their influence, mice lacking pDC have also exhibited a normal influenza disease course, comparable IFNα production and normal viral clearance kinetics following infection with HKx31 H3N2 or PR8 H1N1 IAV [117,127].

In a healthy aging human population (>65 years old with no comorbidities) the circulating myeloid cDC population remains stable [128] with pDC reported to either decrease in frequency with age [128–131] or to not diminish at all [132,133]. Similarly, the influence of age on pDC TLR expression is disputed, with decreased TLR7 expression reported in elderly individuals [128], and comparable expression observed by others [132]. Interestingly, two studies examining moDC from aged individuals (65–88 and 64–92 years old) observed a similar phenotype compared to those derived from younger donors (20–35 and 21–40 years old); however, moDC from older donors were less phagocytic, possessed impaired production of type I and III IFNs and either increased or comparable production of IL-6 and TNFα when stimulated with PR8 IAV or viral elements *in vitro* [133,134]. This age-associated expression defect was linked with epigenetic changes at IFN gene loci, whereas production of IL-6, TNFα, IL-1β, CCL2 and CXCL8 remained unchanged. Levels of IL-1β and IL-18 are decreased in the lungs of HKx31-infected elderly BALB/c mice (15 months of age), which was linked with reduced NLRP3 inflammasome activity in pulmonary DC as well as in bone marrow-derived DC after culture with the virus [23]. Further, adoptive transfer of bone-marrow derived DC from young (2–4 months of age) mice into aged recipients, restored the production of these cytokines following IAV infection and significantly ameliorated IAV-induced morbidity. The elevated basal proinflammatory environment seen in aging individuals, termed inflammaging [135], has been implicated in the defective migration of DC from the lung to draining lymph node during IAV infection. From the age of 6 months, both CD11b^+^ and CD103^+^ cDC display decreased ability to migrate to the lymph nodes following PR8 infection of C57BL/6 mice, which inversely correlated with increasing mouse age and susceptibility to infection [24]. Elevated basal levels of prostaglandin D2 (PGD_2_) in BAL fluid, which can inhibit DC migration, corresponded with the increasing age of C57BL/6 mice and were maintained after IAV infection (Figure 1). PGD_2_ antagonism restored DC migration in PR8-infected aged mice (22-month-old) and correlated both with upregulation of CCR7 on cDC and a significantly enhanced influenza-specific T cell response [24]. Age-related defects in DC migration to the lymph node has also been attributed to cell-intrinsic factors such as reduced phosphoinositide 3-kinases (PI3K) signaling, a positive regulator of DC migration and phagocytosis [133,136].

Most studies agree that type I IFN production is impaired in pDC in older individuals, whereas the production of other inflammatory cytokines and chemokines such as IL-6, IL-10, TNFα and CXCL10 are maintained [128,131,132]. *In vitro* stimulation of young (20–35 years old) compared to aged (65–90 years old) human pDC with IAV led to decreased production of type I IFN in the aged population [128,132]. Comparable to observations made with moDC, Sridharan and colleagues found that elderly human (65–89 years old) pDC isolated from the blood were found to have decreased production of both type I and III IFNs after *in vitro* PR8 IAV stimulation compared to pDC from younger individuals (20–35 years old), yet production of other cytokines such as TNFα and IL-6 was unaffected [132]. The same study reported that pDC TLR expression was maintained with age, which corresponds with observations that IAV recognition and uptake is not impeded in aged pDC [131,132]. Instead, the authors demonstrate that this defect was likely the result of a decreased ability to phosphorylate IRF7, resulting in aged pDC being less potent stimulators of effector T cell responses following exposure to IAV [132]. Overall, aging appears to result in several respiratory DC impairments, which can include decreased abundance, activity and migration, collectively leading to poorer viral clearance and influenza disease outcomes.

### Neutrophils

Neutrophils are the most abundant type of granulocyte and are the chief lung infiltrators following IAV infection, initially attracted and activated by several chemokines and cytokines, particularly CXCR2 ligands, produced by lung-resident cells [137]. In response to IAV infection, lung-infiltrating neutrophils have been shown to destroy infected cells by phagocytosis and to produce reactive oxygen species (ROS), lytic granules and DNA-rich neutrophil extracellular traps (NETs), as well as generate inflammation and promote further leukocyte recruitment, including CD8^+^ T cells and additional neutrophils [92,137–140]. In the context of influenza, neutrophils may be described as a “double-edged sword”, crucial for early protection and control of infectious pathogens, yet also capable of deleterious immunopathology. Depletion of neutrophils in mice prior to infection with a range of H1N1, H3N2 and H5N1 IAV of varying virulence results in worsened weight loss, survival and ARDS-like disease as well as increased viral titers and extrapulmonary viral spread [30,62,89,92,95,139,141,142]. From studies in mice, circulating neutrophils are known to enter the lungs by CXCR2-dependent chemoattraction in two waves following IAV infection with the initial arrivals promoting further neutrophil recruitment [22,95,143]. In contrast to the increased morbidity and mortality exhibited by mice depleted of neutrophils, the complete ablation of neutrophil trafficking to the lung observed following PR8 infection of CXCR2-deficient BALB/c mice had no significant effect on IAV clearance, inflammation, lung tissue damage or survival rate [143]. However, CXCR2 confers responsiveness to a series of chemokines and its expression is not unique to neutrophils, which may account for this contradictory observation [144].

Neutrophils retain the greatest cytotoxic potential of the several leukocyte populations that rapidly infiltrate the lung following IAV infection; however, excessive infiltration and dysregulated activation of neutrophils is closely linked to acute lung injury and lethal disease in infected individuals and mouse models, particularly in response to infection by lethal doses of highly virulent 1918 pandemic and PR8 H1N1, as well as H5N1 IAV [92,95,145]. PR8 IAV-infected mice lacking type I or III IFN signaling more readily succumb to infection and this correlates with increased CXCL1 production and neutrophil accumulation in the lung compared to wildtype C57BL/6 mice [94,121,122,124]. Patients with severe H1N1, H3N2 and H7N9 influenza disease have increased circulating neutrophils and higher levels of IL-6 and CXCL8 in their serum [146,147]. Both human blood and mouse whole lung transcriptomic analysis of IAV-infected individuals and mice, respectively, strongly linked neutrophil activation with severe disease and lethality [95,146,148]. From their transcriptomic analysis, Tang and colleagues determined that high expression of the neutrophil-specific marker CD177 was highly predictive of patient mortality [146]. NETs are an important antimicrobial defense mechanism composed of DNA and various proteins including myeloperoxidase (MPO) and NET release by neutrophils requires MPO activity and ROS generation [138,149,150]. Excessive production of NETs has been implicated in lung injury during PR8 infection of C57BL/6 and BALB/c mice and increased NET-associated gene expression and MPO-DNA complexes have been observed in the patients with severe influenza [92,146–148,151]. Neutrophils taken from the lungs of IAV-infected mice released NETs in response to IAV-infected LA-4 cells, a mouse respiratory epithelial cell line [92] and similarly, NET-containing supernatant from neutrophil cultures of patients with severe H7N9 IAV infection disrupted lipopolysaccharide-primed epithelial cell monolayers *in vitro*, which was mitigated by the addition of DNase [147]. Intriguingly, measured antibody-mediated reduction, but not complete depletion, of neutrophils during, or depletion subsequent to IAV infection was found to either have no profound effect on IAV-induced morbidity and mortality or actually improved IAV infection resistance [30,89,95]. These results suggest that during influenza disease there is a delicate balance of neutrophil activity that could be redressed to limit damaging immunopathology by lung-infiltrating neutrophils, while retaining their contribution to early host defense against the virus.

In general, neutrophils from older individuals exhibit less phagocytic, microbicidal and migration ability [152]; however, how these functional defects affect influenza disease outcomes have not been elucidated. Studies of IAV-infected aged mice have shown significantly higher numbers of granulocytes retained in the lung (Figure 1), potentially caused by impaired clearance of the cells by AM, which also correlates with persistent high levels of IL-6, IL-1β and MPO [22,25,26,142]. The impact of age on neutrophil responses to IAV infection has more recently been thoroughly investigated in aged mice. Kulkarni and colleagues observed neutrophils were markedly more abundant in the lung and blood of older C57BL/6 mice (18–22 months of age) 6 days following PR8 infection [30]. Targeted depletion of neutrophils prior to or at this point post-infection resulted in worse or improved survival during IAV infection, respectively and independent of age, suggesting that the differing roles of neutrophils at different stages of IAV infection is maintained. Although viral titers remained unchanged, proinflammatory cytokines and indicators of lung damage were reduced when neutrophil depletion was delayed. Aged mice had significantly higher levels of TNFα, IL-6, the crucial neutrophil-attracting chemokines CXCL1 and CXCL2, as well as IL-17, which reinforces the production of these chemokines by AEC [30]. Aged macaques infected with H1N1 IAV had increased CXCL8 and IL-6 in the lung which, although not assessed in these studies, could facilitate greater neutrophil accumulation at the site of infection and potentially increase NET release [31]. Whether excessive NET release in the lung significantly contributes to the increased influenza susceptibility observed in elderly individuals is still unclear; however, human neutrophils appear to have an age-associated impairment in NET production after *in vitro* stimulation with CXCL8 and *in vivo* deoxyribonuclease (DNase) treatment to breakdown NETs does not improve IAV-induced lethality in aged mice [30,153].

### Natural killer cells

NK cells are bone-marrow-derived, granular lymphocytes that account for approximately 10% of circulating lymphocytes in humans. NK cells respond rapidly to viral infection by the targeted killing of infected cells via secretion of cytotoxic granules and providing an important early source of antiviral cytokines such as IFNγ, which can also skew the ensuing adaptive T cell response [154,155]. In mice, surface expression of killer cell lectin-like receptor subfamily G member 1 (KLRG1), CD11b and CD27 on NK cells define different maturity states and indicate distinct functionality, with terminally differentiated, highly cytolytic NK cells identified as CD11b^+^ CD27^−^ KLRG1^+^ [156–159]. The heterogenous human NK cell population can be divided similarly; however, they are commonly categorized CD56^bright^ NK cells, which are proficient at cytokine production and the more mature, cytotoxic CD56^dim^ NK cells, which predominate in the blood and lung [156,160–162]. Finally, the lung also houses a small population of NK cells that express markers of tissue residence, including CD69, CD103 and CD49a [162,163].

NK cells proliferate and accumulate in the lung during the first days of IAV infection where they kill IAV-infected cells, produce antiviral type II IFN (IFNγ) and promote regeneration of lung epithelium via secretion of IL-22 [29,164,165]. This includes lung-resident CD49a^+^ NK cells, which can rapidly respond to IAV infection of human lung explants [163]. Human and murine NK cells are susceptible to A/Hong Kong/54/98 H1N1 and PR8 H1N1 IAV infection, respectively, which leads to functional impairment of the cells and induces programmed cell death, which thwarts viral dissemination [166,167]. Type I and II IFN signaling is crucial for NK cell activity following PR8 infection as NK cells in *Ifnar1^-/^*^-^ mice failed to produce IFNγ or degranulate. IFNγ administration promptly after PR8 infection of mice increased lung NK cell numbers, cytotoxicity and improved resistance to infection, which was lost when NK cells were depleted [168,169]. The latter observation is congruent with several additional studies using antibody-mediated NK cell depletion with either anti-NK1.1 or anti-asialo-GM1, suggesting a critical role for NK cells in early defense against IAV infection [15,29,170–172] and corresponds with consistent observations of NK lymphopenia in severe 2009 H1N1 pandemic infections in humans [173,174]. Conversely, others have shown that NK cells can contribute to influenza-associated pathology and decreased survival using similar *in vivo* NK cell depletion methods but seemingly only during lethal PR8 infection [175,176]. Care must be taken when interpreting these results, however, as the common depleting antibody targets NK1.1 and asialo-GM1 are expressed on other cells such as pulmonary macrophages, NKT cells, CD8^+^ T cells [177–179], including activated influenza-specific CD8^+^ T cells in the lung [180]. Interestingly, others have recently reported improved survival rates and reduced immunopathology in IFNγ-deficient mice infected with 2009 pandemic H1N1 IAV [106,181]. Importantly, NK cells can eliminate infected cells by recognizing the IAV HA via the activation receptor NKp46 and mice lacking this receptor are more susceptible to IAV infection [182–184]. The same receptor is necessary for NK-cell-mediated apoptosis of neutrophils, which may be important for the resolution of inflammation within the lung following IAV infection [185].

Several age-associated changes to NK cells have been observed in both mice and humans (Figure 1). At the steady state aged (22-month-old) C57BL/6 mice have reduced frequency and absolute numbers of lung NK cells [186]. Furthermore, NK cells in the lung and spleen of aged mice expressed fewer markers of maturity, potentially resulting from impaired NK cell production in the bone marrow [186,187]. In humans, aging is associated with a profound reduction in the ratio of circulating CD56^bright^ to CD56^dim^ NK cells, an increase in the frequency of NK cells bearing the maturation marker CD57, either comparable or reduced NK cell activity, including decreased IFNγ and CXCL8 secretion, a slower rate of homeostatic NK cell generation and finally, fewer NK cells expressing NKp46, a key receptor in defense against IAV spread and disease resolution [183,185,188–191]. Reduced NK cell abundance and maturation in older mice does not appear to be due to intrinsic defects but instead is a consequence of the aged environment, as donor bone marrow or splenic NK cells from both young (4–6 months) or aged (20–22 months) mice displayed improved development, proliferation and upregulation of KLRG1 when transferred into young recipients but not older ones [192]. The majority of evidence of age-associated changes in NK cell responses to influenza comes from aged mouse models of infection. Although no significant differences in NK cell activity was evident in young (6–8 weeks) and aged (22 months) C57BL/6 mice at the steady state, Nogusa and colleagues observed that following PR8 infection, NK cells failed to expand in either the lung or spleen of aged mice, with the former also displaying reduced cytotoxicity [29]. Interestingly, a later study from the same group observed fewer NK cells in the lungs and spleen of naïve and PR8 IAV-infected young (6 months) and aged (22 months) C57BL/6 mice, which were also less cytotoxic, less responsive to NKp46 stimulation and were comprised of more immature CD11b^−^ and less terminally-differentiated CD11b^+^ CD27^−^ NK cells [193]. Age-associated NK cell impairment has also been linked to diet, with mortality and morbidity of aged C57BL/6 mice (>23 months of age) infected with PR8 found to significantly worsen when fed a caloric restricted diet, which was also associated increased viral titers and further decreased lung NK cell cytotoxicity compared to aged control mice [28].

### Type 2 innate lymphoid cells (ILC2)

ILCs are a heterogeneous collection of lymphocytes, including NK cells, that are classified into several subpopulations based on their cytokine expression repertoire, akin to CD4^+^ effector T cells. While NK cells are grouped together with IFNγ-producing type 1 ILC (ILC1), ILC2 are the predominant ILC subset resident in the mouse lung and are characterized by their ability to secrete type 2 cytokines such as IL-4, IL-5, and IL-13 in response to DAMPs such as IL-25 and IL-33 [194–198]. Although ILC1, ILC2 and the IL-17-producing ILC3, can all be found within the lung [199], the majority of research in the context of IAV infection has concentrated on ILC2 [200,201]. In mice and humans, the phenotype of lung ILC2 can vary, yet they are typically identified by a lack of common immune cell lineage markers and their expression of a combination of CD25, CD127, CD90, KLRG1 and ST2, the receptor for IL-33^64,^ [202,203]. IL-33 is produced by pulmonary epithelial and endothelial cells, AM and natural killer T (NKT) cells following infection with A/WSN/1933 H1N1, as well as A/Memphis/71 H3N2 IAV and in response, ILC2 proliferate and can exacerbate airway hyperreactivity in an IL-13-dependent manner in the absence of T cells [86,87,204].

Alternatively, ILC2 can also play a beneficial role in the context of IAV infection. Following infection of C57BL/6 mice with HKx31 H3N2, PR8 or 2009 pandemic H1N1, ILC2 expand, peaking around day 4 post-infection and persist in the lung until the later stages of infection to support lung function, tissue integrity and tissue remodeling, the latter being critical for lung repair and tissue homeostasis [64–66]. Adoptive transfer of ILC2 improved influenza-associated morbidity and required IL-33/IL-33 R signaling and the growth factor, amphiregulin [64]. Using IFNγ-deficient mice and anti-IFNγ neutralization, Califano and colleagues demonstrated that ILC2 promote increased resistance to 2009 pandemic H1N1 via the production of amphiregulin and IL-5 activity, which is ordinarily under IFNγ-mediated suppression [65]. These findings were congruent with previous observations that ILC2 responses, including their production of IL-5 is negatively regulated by type I IFNs and IL-27 in a signal transducer and activator of transcription 1 (STAT1)-dependent manner and that IL-33-induced airway hyperreactivity was ameliorated by the IFN-γ supplementation [205,206]. Fewer mature ILC2 populate the lungs of aged mice (19–24 months of age) and have diminished functionality compared to younger counterparts (2–3 months of age) (Figure 1), which may be partially due to changes in the lung microenvironment, as indicated by elevated levels of the proinflammatory cytokines IL-18 and IL-12 [27]. This numerical disparity was sustained following 2009 pandemic H1N1 infection, which resulted in greater lung inflammation and complete lethality of the older mice [Intriguingly, only adoptive transfer of lung-resident ILC2 from IL-33-treated young mice into aged recipients, lessened infection-induced weight loss and lung inflammation and significantly improved the survival rate of older mice.

## Conclusion

Aging influences the cells of the innate immune system in multiple different ways and to varying degrees. Continued examination of the intrinsic and extrinsic age-related factors that induce numerical and functional changes in innate immune cells will be crucial to inform the design of new vaccines, adjuvants, supplements and drugs to improve outcomes for the elderly, who account for the majority of influenza-associated hospitalizations and deaths. As IAV infection susceptibility is often disconnected from viral titer, this could be facilitated by exploiting elements of the immune system to bolster influenza disease tolerance and recovery in the elderly, rather than direct targeting of the virus with antiviral drugs that show limited efficacy. For example, adjusting the inflammatory environment within the IAV infected lung, which may already be dysregulated in an elderly patient, could reduce potential immunopathology, the chance of secondary bacterial infections and expedite disease resolution. In addition to the innate immune cells described above, there are multiple other immune cells with emerging roles in IAV infection that display some degree of immunosenescence or age-associated alteration that could be manipulated. For example, unconventional T cells that encompass NKT cells, gamma delta T cells and mucosal-associated invariant T cells, share features of innate and adaptive immunity, being able to respond to both antigen-specific and cytokine stimuli [207]. Although the cognate antigens for these cells are not necessarily produced during IAV infections, unconventional T cells can rapidly respond to induced proinflammatory cytokines such as IL-18 and IL-12, which can be elevated with aging, and release a wide array of cytokines that can influence the succeeding adaptive immune response and therefore the course of the disease [27,207–210]. Therefore, unconventional T cells as well as the innate immune cells highlighted in this review that significantly contribute to IAV immunity and recovery from IAV infection, warrant further investigation in this setting.

Currently, most of our knowledge on the effects of aging on immunity to IAV stems from mouse models, which have several notable caveats highlighted throughout this review, that need to be considered when attempting to translate any findings to the clinic. The inbred laboratory mice used in most of the studies reviewed here lack functional Mx1, an IFN I and III–inducible antiviral protein that limits IAV replication. Fully IFN-competent Mx1-sufficient mice may serve as a more faithful model of the early antiviral response to IAV infection and depending on the genetic background, can exhibit greater resistance to IAV infection. As Mx1 gene expression diminishes with age, future studies on the influence of aging on IAV pathogenesis, vaccination or candidate therapeutics would ideally be conducted with aged Mx1-sufficient mice and potentially newly developed humanized mice engrafted with human immune systems, prior to extension to higher order animals and humans [18]. Another potential consideration is the frequent use of the PR8 strain of IAV, which was adapted to mice by >300 sequential passages in mouse lung [211]. PR8 is likely to have acquired mutations that facilitate evasion of host responses and as such PR8 poorly infects murine respiratory macrophages, the sentinels of the respiratory tract and is highly virulent [43,44]. Moreover, the fate of murine AM following IAV infection is seemingly dependent on mouse genetic background, therefore experimental results using PR8 may not accurately reflect human influenza pathogenesis [106]. Lastly, use of less virulent IAV such as HKx31 H3N2 at varying inoculum doses to model mild and severe disease may be more informative in aged animal models of IAV infection, particularly as elderly IAV-associated mortality is highest during seasonal epidemics dominated by the H3N2 subtype [1].

The heightened baseline inflammatory environment that corresponds with aging (inflammaging) influences innate immune cell responses, typically manifesting as altered activity and abundance within the infected lung and resulting in less efficient resolution and recovery from IAV infection in older organisms. With increased age, alveolar epithelial cells (AEC) appear more senescent, regenerate slower and secrete increased levels neutrophil-attracting chemokines upon IAV infection. AM are less numerous, poorly phagocytic and produce higher levels of CXCL8, favoring further recruitment and persistence of neutrophils in the airways. Similarly, elevated levels of CCL2 in the infected lungs facilitate increased entry of monocyte-derived Ly6C^hi^ inflammatory macrophages from the circulation. Respiratory conventional and monocyte-derived dendritic cells (cDC and moDC) secrete cytokines in the lung and traffic antigen to draining lymph nodes via the lymphatics. Elevated levels of prostaglandin D_2_ (PGD_2_) in aged mice contributes to impaired cDC migration to the lymph node and T cell responses and moDC have an age-associated defect in type I and III IFN production. pDC display a similar defect in IFN production, which correlates with reduced expression of TLR7, a sensor of viral ssRNA. Aging is linked to reduced NK cell terminal differentiation, expansion, cytotoxicity and NKp46-mediated recognition of IAV-infected cells and fewer type 2 innate lymphoid cells (ILC2) populate the lungs in aged mice and are functionally impaired. In the context of IAV infection, ILC2 respond to the damage-associated molecular pattern IL-33 and can support lung tissue integrity, remodeling and repair.

## Data Availability

Data sharing is not applicable to this article as no new data were created or analyzed in this study.
